# On the Structure of Ultrathin FeO Films on Ag(111)

**DOI:** 10.3390/nano8100828

**Published:** 2018-10-13

**Authors:** Mikołaj Lewandowski, Tomasz Pabisiak, Natalia Michalak, Zygmunt Miłosz, Višnja Babačić, Ying Wang, Michał Hermanowicz, Krisztián Palotás, Stefan Jurga, Adam Kiejna

**Affiliations:** 1 NanoBioMedical Centre, Adam Mickiewicz University, Umultowska 85, 61-614 Poznań, Poland; zmilosz@amu.edu.pl (Z.M.); visbab@amu.edu.pl (V.B.); yinwan@amu.edu.pl (Y.W.); stjurga@amu.edu.pl (S.J.); 2 Institute of Experimental Physics, University of Wrocław, Pl. M. Borna 9, 50-204 Wrocław, Poland; adam.kiejna@uwr.edu.pl; 3 Institute of Molecular Physics, Polish Academy of Sciences, M. Smoluchowskiego 17, 60-179 Poznań, Poland; michalak@ifmpan.poznan.pl; 4 Institute of Physics, Poznan University of Technology, Piotrowo 3, 60-965 Poznań, Poland; michal.hermanowicz@put.poznan.pl; 5 MTA-SZTE Reaction Kinetics and Surface Chemistry Research Group, University of Szeged, 6720 Szeged, Hungary; kpalotas77@gmail.com

**Keywords:** iron oxides, ultrathin films, silver, epitaxial growth, structural characterization, STM, LEED, XPS, DFT, model system

## Abstract

Ultrathin transition metal oxide films exhibit unique physical and chemical properties not observed for the corresponding bulk oxides. These properties, originating mainly from the limited thickness and the interaction with the support, make those films similar to other supported 2D materials with bulk counterparts, such as transition metal dichalcogenides. Ultrathin iron oxide (FeO) films, for example, were shown to exhibit unique electronic, catalytic and magnetic properties that depend on the type of the used support. Ag(111) has always been considered a promising substrate for FeO growth, as it has the same surface symmetry, only ~5% lattice mismatch, is considered to be weakly-interacting and relatively resistant to oxidation. The reports on the growth and structure of ultrathin FeO films on Ag(111) are scarce and often contradictory to each other. We attempted to shed more light on this system by growing the films using different preparation procedures and studying their structure using scanning tunneling microscopy (STM), low energy electron diffraction (LEED) and X-ray photoelectron spectroscopy (XPS). We observed the formation of a previously unreported Moiré superstructure with 45 Å periodicity, as well as other reconstructed and reconstruction-free surface species. The experimental results obtained by us and other groups indicate that the structure of FeO films on this particular support critically depends on the films’ preparation conditions. We also performed density functional theory (DFT) calculations on the structure and properties of a conceptual reconstruction-free FeO film on Ag(111). The results indicate that such a film, if successfully grown, should exhibit tunable thickness-dependent properties, being substrate-influenced in the monolayer regime and free-standing-FeO-like when in the bilayer form.

## 1. Introduction

Ultrathin films grown on single crystal supports are believed to constitute a new class of 2D materials with properties governed by low-dimensionality and the interaction with the substrate and, therefore, different from those of the corresponding bulk materials [1]. The substrate- and thickness-mediated effects are particularly pronounced in the case of ultrathin films of insulators or semiconductors grown on conducting supports, where the overlapping of the electronic states of the film and the substrate takes place. Historically, such films were meant to be used as model systems that would allow the use of surface science tools for the studies of materials that are not electrically conductive in the bulk form [2]. However, the unpredicted at the time film-substrate interactions, often manifested by structural reconstructions, such as Moiré superstructures, were found to increase the complexity of those systems and limit their initially intended application. Quite unexpectedly, the unique properties of ultrathin films opened new pathways for the rediscovery of the well-known simple compounds, such as transition metal oxides or dichalcogenides, in their unnatural two-dimensional form, and many reports describing such systems have been published to date.

Monolayer iron oxide (FeO) films can be grown in the close-packed <111> direction on various (111)-oriented and (0001)-oriented metal single crystal supports [3,4,5,6,7]. To date, all such films were found to exhibit lattice mismatch-induced Moiré superstructures which give them unique properties but also make none of them fully-representative as model system imitating bulk oxide material (which would be also interesting to achieve). Among the substrates used, Ag(111) has always been considered a promising candidate for iron oxides growth, as it has the same surface symmetry as FeO(111), only ~5% lattice mismatch (much smaller than the commonly used Pt(111) [3,4]), is considered a weakly-interacting substrate and is relatively resistant to oxidation. There have been several reports on the growth of ultrathin and thin iron oxide films on differently oriented silver single crystal supports [8,9,10,11,12,13,14,15,16,17,18,19]. An overview of these works is presented in the Supplementary Materials file, Section 1 (SM1). Regarding ultrathin FeO(111) films on Ag(111), most reports were contradictory to each other and lacked detailed structural characterization by atomic-resolution techniques, such as scanning tunneling microscopy (STM). The very first work by Waddil and Ozturk suggested that deposition of submonolayer amounts of iron onto a silver substrate kept at room temperature (RT) and subsequent oxidation results in a (1×1) growth of FeO, i.e. adaptation of the surface lattice constant of FeO (3.04 Å) to Ag (2.89 Å) [8]. The assumption was based on low energy electron diffraction (LEED) and X-ray photoelectron spectroscopy (XPS) results. More recently, Lundgren, Weaver and co-workers grew the films using different procedure, i.e. by depositing iron onto a slightly heated silver substrate and in an oxygen ambient [17,18,19]. The authors employed STM and observed the formation of a p(9×9) Moiré superstructure with a significantly expanded FeO surface lattice constant (to 3.25 Å) and very small separation between iron and oxygen layers (0.26 Å—meaning that the iron and oxygen atoms are almost in-plane). The superstructure was also visible on the acquired LEED patterns. The conclusions on the structure of FeO(111)/Ag(111) drawn by the two groups differ, however, it has to be underlined that (1) the preparation procedures used by both groups were not the same, (2) the authors of Ref. [8] did not employ STM (or any other atomic-resolution technique) to determine the exact structure of their films and (3) the authors of Refs. [17,18,19] were clearly stating that their films are not uniform and consist of a mixture of well-defined Moiré-reconstructed FeO (majority phase) and ill-defined “FeO_x_” species (minority phase).

We try to shed more light on the structure of ultrathin FeO films grown on Ag(111). In our experiments, we applied different preparation procedures and used STM, LEED and XPS to analyze the structure of the fabricated films. Firstly, we deposited iron onto the silver substrate kept at room temperature and post-oxidized it. We found that such procedure leads to the growth of Moiré-reconstructed and structurally ill-defined islands located mainly at the silver step edges. Interestingly, the structural parameters of the observed Moiré superstructure differ significantly from those reported in References [17,18,19]. Secondly, we modified the procedure by depositing iron onto a heated silver substrate and post-oxidizing. In that way we succeeded to grow uniformly distributed surface structures, some of which were reconstructed and some reconstruction-free. Some of the observed reconstructed regions were virtually identical to those observed following room-temperature iron deposition and oxidation. The nature of the reconstruction-free regions was difficult to identify unambiguously, however, their atomic structure and electronic characteristics seemed to differ from those of clean Ag(111). We also performed density functional theory (DFT) calculations on the structure and properties of a conceptual reconstruction-free FeO/Ag(111). The results indicate that such film, if successfully grown, should exhibit tunable thickness-dependent properties, being substrate-influenced in the monolayer regime and free-standing-FeO-like when in the bilayer form.

## 2. Experimental and Theoretical Methods

The experiments were performed in an ultra-high vacuum (UHV) system (from Omicron, Taunusstein, Germany) consisting of three inter-connected chambers: The preparation chamber (base pressure: 5 × 10^−10^ mbar), the scanning probe microscopy chamber (base pressure: 3 × 10^−11^ mbar) and the load-lock chamber. The preparation chamber hosted a cold cathode sputter gun, single electron beam evaporator and e-beam heating stage. It was also equipped with a LEED instrument and an XPS setup. The scanning probe microscopy chamber was equipped with a variable-temperature STM. All STM measurements were performed in a constant current mode using W tips. The Ag(111) single crystal (purity: 99.999%, polishing accuracy: <0.1°; from MaTeck GmbH, Jülich, Germany) was cleaned by repeated cycles of 1 keV and 0.6 keV Ar^+^ (purity: 99.999%; Messer Industriegase GmbH, Bad Soden, Germany) ion sputtering, annealing in O_2_ (purity: 99.999%; Messer Industriegase GmbH, Bad Soden, Germany) and under UHV at T ≥ 700 K. Iron (purity: 99.995%; Alfa Aesar GmbH, Karlsruhe, Germany) was evaporated from a 2 mm rod onto a Ag(111) substrate kept at RT or 500–600 K. The deposition rate, calibrated using STM, was approx. 2 monolayers (MLs) per minute (calibration accuracy: 10%), where 1 ML is defined as the amount of iron that would cover the Ag(111) surface with a closed bcc Fe(110) film. The STM images were processed using Gwyddion (ver. 2.4x, Open Source) [20] and WSxM (ver. 5.0 Develop 8.x, Freeware) [21] computer software. The oxidation of iron was performed by backfilling the preparation chamber with molecular oxygen using a leak valve and heating the sample to 700 K. Following 15–30 min oxidation, the sample was cooled down in oxygen for several minutes. The temperature of the sample was measured using an infrared pyrometer focused on a tantalum holder on which the sample was mounted. The cleanliness of the substrate, as well as the structure of Fe and FeO deposits, were characterized by STM, LEED and XPS. Scanning tunneling spectroscopy (STS) dI/dz experiments were performed using a lock-in technique: A modulation voltage of 60 mV at a frequency of 6777 Hz was applied to the z-piezo of the STM (which resulted in a periodic tip-sample distance change of +/− 0.1 nm), the STM bias voltage was set to 50 mV and the curves were acquired locally from the objects of interest. For each measurement, the tip was first retracted from the sample’s surface by 1 nm (to attenuate any tip-sample interactions) and then brought to the surface with a speed of 1 nm/s. Each of the presented curves is a result of averaging of 15 similar measured curves and additional smoothing using the locally weighted scatterplot (LOESS) method. The LEED patterns were recorded at various energies ranging from 30 eV to 255 eV. The XPS measurements were performed using a monochromatic AlKα (1486.6 eV) X-ray source and a semispherical electron energy analyzer operating at a pass energy of 50 eV (survey) and 20 eV (regions). The data were calibrated with respect to the Ag 3d_5/2_ peak (368.2 eV) [22] and fitted using CasaXPS computer software (ver. 2.3.19PR1.0, Casa Software Ltd, Teignmouth, UK). A linear combination of Gauss and Lorentz functions (the so called Voigt function) and Shirley background subtraction were used for the fittings. Detailed information on all XPS fittings presented in this work is provided in SM2.

The DFT calculations were performed using the Vienna ab initio simulation package (VASP, ver. 5.4.4, Vienna, Austria) [23,24,25]. The electron-ion core interactions were represented by the projector-augmented wave (PAW) potentials [26,27], with Ag 4d^10^5s^1^, Fe 3d^7^4s^1^ and O 2s^2^2p^4^ states considered as the valence states. A plane wave basis set with a kinetic energy cutoff of 400 eV was applied. The exchange-correlation energy was treated at the spin-polarized generalized gradient approximation (GGA) level using the Perdew-Burke-Ernzerhof (PBE) functional [28]. The Brillouin zone was sampled using Γ-centered k-point meshes. A Fermi surface broadening of 0.2 eV was applied to improve convergence of the solutions using the second order Methfessel-Paxton method [29]. To account for the strongly correlated 3d electrons localized on the Fe ions, the Hubbard U correction was applied (GGA+U) within the rotationally invariant approach of Dudarev et al., with the effective parameter U_eff_ = U − J = 3.0 eV, where (U,J) = (4,1) eV are the Coulomb and screened exchange parameters, respectively [30]. The applied U_eff_ value is known to provide a satisfactory description of bulk characteristics of FeO [31]. Convergence threshold for the total energy of the studied systems was set to 10^−6^ eV. The lattice parameter of bulk fcc Ag, a = 4.155 Å, calculated using 16×16×16 k-points mesh, was found to agree well with other GGA calculations performed using a similar computational method (e.g. 4.16 Å [32] or 4.14 Å [33]) and overestimated the experimental value, 4.086 Å [34], by less than 1.7%. A silver (111) substrate was modelled with an asymmetric slab consisting of four atomic layers of Ag with a 1×1 or 2×2 surface unit cell. In order to check the appropriateness of the size of the unit cell, additional calculations for larger supercells were also performed. The slabs were separated from their periodic images by a vacuum region of 18 Å. A Γ-centered 16×16×1 k-point mesh was applied for the surface 1×1 unit cell and appropriately reduced for larger cells. The positions of atoms in the bottom two Ag layers were frozen and the atomic positions of the remaining atoms were optimized until the residual Hellman-Feynman forces on atoms were smaller than 0.01 eV/Å. The optimization of the bare surface structure yielded negligibly small expansion of the topmost interplanar separation and a 0.8% contraction of the second interlayer distance. The work function was calculated as the difference between the electrostatic potential energy in the vacuum region and the Fermi energy of the slab. A dipole correction was applied to compensate for the asymmetry of the slab and obtain correct work function values [35]. The calculated work function of the clean Ag(111) surface, 4.49 eV, is very close to the experimental value of 4.46 ± 0.02 eV [36], and the value obtained in other calculations, 4.50 eV [37], using a similar computational method. FeO(111) monolayer was adsorbed on one side of the Ag(111) slab. For the initial structural modelling, the calculated in-plane lattice constants of 2.938 Å for Ag(111) (PBE) and 3.032 Å for FeO(111) (PBE+U) were used [38]. The system was re-optimized after the deposition of a FeO layer within the applied surface unit cell. The electron charges on atoms were calculated using Bader analysis [39,40]. The STM images were simulated using the Tersoff-Hamann method [41].

## 3. Results and Discussion

### 3.1. Iron Deposition at Room Temperature and Post-Oxidation

**Figure 1:**
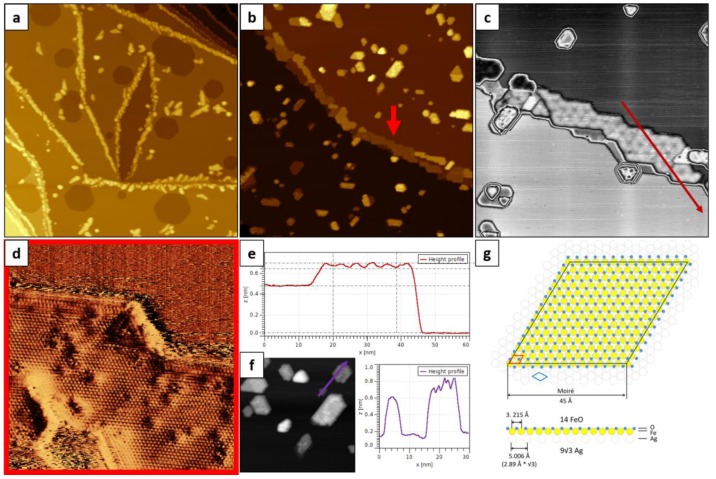
Large-scale topographic scanning tunneling microscopy (STM) images (300 × 300 nm^2^) of 0.5 ML Fe deposited onto Ag(111) substrate kept at room temperature (**a**) and subsequently oxidized in 1 × 10^−6^ mbar O_2_ at 700 K (**b**). (**c**) and (**d**) are zoom-in images of (b) (100 × 100 nm^2^ and 20 × 20 nm^2^, respectively) showing the Moiré superstructure (c) and the atomic structure (d) of the reconstructed island (marked with red arrow in (b)). (**e**) presents the height profile marked in (c). (**f**) shows an STM image (75 × 75 nm^2^) of islands with ill-defined structure, as well as an exemplary height profile of such islands (marked on the neighboring STM image). (**g**) presents the proposed model for the observed Moiré superstructure, where the blue rhombus marks the Ag(111) surface cell, the red one the FeO(111) cell and black the Moiré cell (see text for details). Tunneling parameters: +0.7 V (all images), 0.4 nA (a,d,f) and 1.0 nA (b,c).

Firstly, we deposited Fe onto a Ag(111) substrate kept at room temperature and post-oxidized it. As prepared iron/silver and iron oxide/silver samples will be further referred to as RT-Fe/Ag(111) and RT-FeO/Ag(111), respectively. Figure 1a presents a large-scale STM image of the 0.5 ML RT-Fe/Ag(111) sample. The deposited iron mainly agglomerated at the silver step edges forming irregularly-shaped nanowires (such growth has also been observed by other authors, see e.g. Reference [42]). In addition, the deposition was found to lead to silver steps erosion and vacancy islands formation (similar substrate etching has been reported for Cu/Ag(111) [43]).

Typical structures observed after oxidation, together with their height profiles, are shown in different magnification in Figure 1b–e. Irregular iron particles mostly changed into well-defined hexagonally-shaped islands (Figure 1b–d). Interestingly, the orientation of the island edges indicated that the <110> crystallographic direction, characteristic for the step edges of (111)-oriented surfaces [44], is not preferred for the as grown FeO islands, while the $\sqrt{3}$ (<112>, <121>, <211> and equivalent) directions are. On most islands, a Moiré superstructure was observed (Figure 1b,c, marked with a red arrow). The atomic resolution image of such an island, shown in Figure 1d, revealed a close-packed atomic arrangement with ~3.2 Å periodicity, while its line profile, presented in Figure 1e, provided information on the height of a typical Moiré-reconstructed island (~2.1 Å), the Moiré period (45 Å) and the Moiré corrugation (0.4–0.5 Å). Occasionally, a second or even third layer of FeO was observed, with the height of one layer always being around 2.1 Å. The Moiré structure itself was found to run along the $\sqrt{3}$ crystallographic directions of the support (similarly to the island edges). The observed atomic periodicity and height indicated that the islands are indeed FeO(111), as other iron oxide phases (i.e. Fe_3_O_4_(111) or Fe_2_O_3_(0001)) would show much larger atomic periodicities in STM (6 Å and 5 Å, respectively [4]), as well as larger heights. The observed height and Moiré corrugation may, of course, depend on the tunneling parameters and tip condition, which are of particular importance when measuring height differences between oxide and metal surfaces. For instance, on the atomic-resolution STM image shown in Figure 1d no Moiré corrugation is visible. The height profile taken along exemplary islands with ill-defined surface structure is presented in Figure 1f. These islands were mainly located at the regular terrace sites of silver, were much higher than the Moiré-reconstructed islands and had a disordered surface structure.

The proposed model for the observed Moiré superstructure is presented in Figure 1g. Based on the Moiré and atomic periodicities, as well as the orientation of the island edges and the Moiré itself, we conclude that the coincidence structure is a 30°-rotated 14×14 FeO on 9×9$\sqrt{3}$ Ag. The numerically determined FeO lattice constant for such a superstructure (3.215 Å) and the Moiré period (45 Å) are in perfect agreement with the experimentally measured values.

The most important finding is that the structural parameters of the observed Moiré superstructure differ significantly from those reported by Lundgren, Weaver and co-workers for the Moiré-reconstructed FeO/Ag(111) (26 Moiré period, 3.25 Å atomic period and no Moiré rotation with respect to the support) [17,18,19]. This contradiction indicates that different preparation procedures may lead to the formation of different well-ordered surface structures that are energetically the most stable for the particular growth conditions. Such behavior was not observed for FeO(111) on any other close-packed single-crystalline substrate.

**Figure 2:**
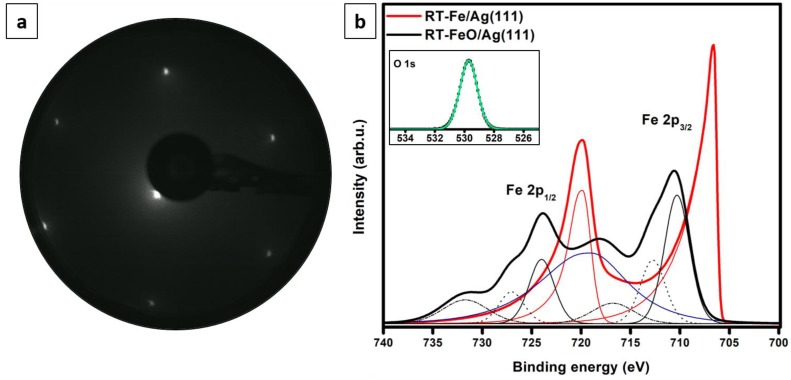
Low energy electron diffraction (LEED) pattern (60 eV) of 0.5 ML FeO film grown by Fe deposition onto Ag(111) substrate kept at room temperature and post-oxidation (**a**). (**b**) shows Fe 2p X-ray photoelectron spectroscopy (XPS) spectra obtained for the 1.0 ML RT-Fe/Ag(111) (red line) and 1.0 ML RT-FeO/Ag(111) (black) samples. The Ag 3s signal, overlapping with the Fe 2p line, is marked in blue. Inset in (b) presents the O 1s spectrum (green line) recorded for the oxidized sample.

The LEED pattern obtained for the oxidized sample shown in Figure 1b is presented in Figure 2a. Due to the agglomeration of the material at the silver step edges and a sole fraction of the substrate exposed, the observed pattern could be still roughly interpreted as (1×1). It is worth noting that a (1×1) LEED was also reported by the authors of Ref. [8] following 1–3 MLs Fe deposition at room temperature and post-oxidation. In contrast, a satellite pattern originating from a 26 Å Moiré-reconstructed FeO was reported by other authors following Fe deposition onto a heated substrate and in an oxygen ambient [17,18,19]. The lack of additional spots on our pattern could be due to small iron oxide coverage (0.5 ML, with a fraction of islands being Moiré-reconstructed and a fraction being structurally ill-defined). Interestingly, a simple simulation of the observed superstructure (Figure 1g) did not produce a clearly-visible Moiré superstructure. Thus, it may be speculated that the experimentally observed corrugation may result from the electronic film-substrate interaction (the lack of visible corrugation on the atomic-resolution image in Figure 1d seems to confirm this theory). The absence of additional spots also confirms the lack of significant amounts of iron oxide phases other than FeO(111), i.e. Fe_3_O_4_(111) or Fe_2_O_3_(0001), that would give a (2×2) and ($\sqrt{3}$×$\sqrt{3}$)R30° LEED patterns, respectively [4,13,15].

The XPS data recorded for the 1.0 ML RT-Fe/Ag(111) and 1.0 ML RT-FeO/Ag(111) samples are presented in Figure 2b. The main graph presents the Fe 2p lines obtained for both samples, while the inset shows the O 1s signal obtained for the oxidized sample. A detailed analysis of the Fe 2p data was not trivial, as the region overlaps with the Ag 3s peak centered at around 719 eV.

The Fe 2p signal obtained for the RT-Fe/Ag(111) sample could be fitted with two components centered at around 706.5 eV and 719.9 eV, which are characteristic of metallic iron and result from spin-orbit coupling (Fe 2p_3/2_ and 2p_1/2_ peaks [45]). The spectrum obtained for the oxidized sample could be fitted with six components—710.3 eV, 712.6 eV, 716.6 eV, 724.0 eV, 727.0 eV and 731.5 eV—which is typical for the FeO phase [46,47]. The 710.3 eV and 724.0 eV peaks, chemically shifted by approx. 4 eV to higher binding energies with respect to metallic iron, result from Fe^2+^ ions in iron oxide. X-ray photoelectron lines of iron oxides, and ionic compounds in general, exhibit broadening due to a multiplet splitting effect (coupling between core holes with unpaired electrons in the outer shell [48,49,50,51]) which, in our case, was manifested by the presence of the components centered at around 713.4 eV and 726.9 eV. The 718.8 eV and 731.0 eV peaks on the other hand, correspond to characteristic Fe^2+^ satellite peaks (which appear as a consequence of photoelectrons exciting valence electrons into unoccupied orbitals [46,51,52]). A binding energy difference of nearly 6 eV between the first satellite and the main Fe 2p_3/2_ photoemission line and of nearly 7 eV between the second satellite and the main Fe 2p_1/2_ line provide clear evidence for the presence of Fe^2+^ iron [46,47,52] (the presence of Fe^3+^ iron would result in a binding energy difference of more than 8 eV [46,52]). Based on the peak positions it may be, therefore, concluded that metallic iron and iron in the Fe^3+^ oxidation state are either not present or present in the amount that falls beyond the detection limit of our XPS system. The oxygen O 1s signal recorded for the oxidized sample could be fitted with a single peak centered around 529.7 eV [49]. The Fe to O ratio, determined from the survey spectrum (not shown) and by taking the elemental photoionization cross-sections [53] into account, was approx. 1:1—as expected for a stoichiometric FeO phase. No other elements, like e.g. carbon, sulfur or other contaminants, could be detected. All these indicated that the observed islands—no matter if well- or ill-defined—consist of Fe^2+^ and O^2−^ ions (however, a contribution of Ag atoms in these structures could not be excluded as well).

### 3.2. Iron Deposition at High Temperature and Post-Oxidation

**Figure 3:**
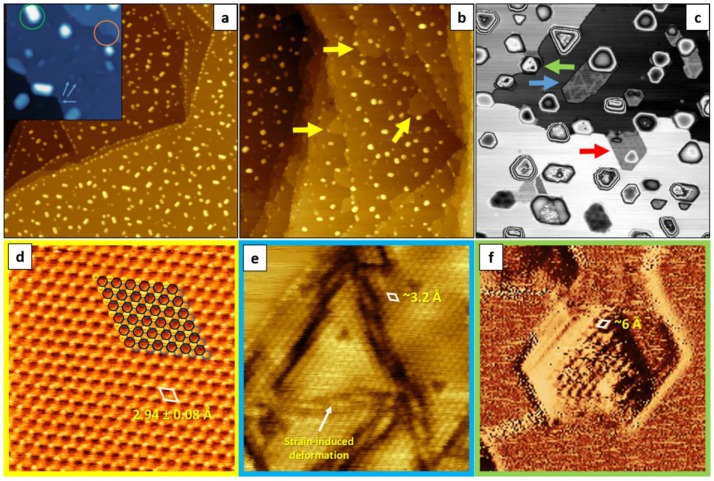
Large-scale topographic STM images (300 × 300 nm^2^) of 0.5 ML Fe deposited onto Ag(111) substrate kept at 550 K (**a**) and subsequently oxidized in 1 × 10^−6^ mbar O_2_ at 700 K (**b**). The inset in (**a**) (50 × 50 nm^2^) shows Fe inclusions in Ag(111) at regular terrace sites (marked with blue arrows) and step edges (orange circle), as well as bcc Fe(110) crystallite (green circle) observed on low-coverage HT-Fe/Ag(111) sample. The yellow arrows in (b) show the reconstruction-free islands. (**c**) shows small-scale image (100 × 100 nm^2^) with various reconstructed surface regions (marked with green, blue and red arrows). (**d**), (**e**) and (**f**) show atomically-resolved STM images of the structures marked with colors in (b) and (c) (5 × 5 nm^2^, 10 × 10 nm^2^ and 15 × 15 nm^2^, respectively). The unit cells are marked with white rhombuses (see text for details). Tunneling parameters: +0.7 V (a,b,c,e,f, inset in (a)) and +0.1 V (d), 1.0 nA (a) and 0.4 nA (b,c,d,e,f, inset in (a)); (d)—sum of topography and current images, (f)—current image.

Secondly, we modified the preparation procedure by depositing Fe onto a 550 K-heated Ag(111) substrate and post-oxidizing it. The corresponding iron/silver and iron oxide/silver samples will be further referred to as HT-Fe/Ag(111) and HT-FeO/Ag(111) (underlining the fact that iron was deposited at high temperature (HT)). Figure 3a,b presents large-scale STM topography images of the HT-deposited 0.5 ML Fe on Ag(111) before (a) and after (b) oxidation in 1 × 10^−6^ mbar O_2_ at 700 K. In contrast to the observed agglomeration of iron at the silver step edges following RT deposition, the HT deposition was found to lead to the formation of well-defined bcc Fe(110) crystallites uniformly distributed over the silver surface. We explain this difference in the growth mode by partial incorporation of iron into the top silver layer at the initial stage of deposition and, thus, formation of nucleation centers for the growing crystallites. This assumption is based on the observation of small, elongated, rhomboidal particles embedded in the top layer of silver following the deposition of submonolayer iron amounts. The examples of such inclusions are shown in the inset to Figure 3a (marked with blue arrows); they have a height of ~0.6–1.2 Å (approx. half of the height expected for monolayer bcc-Fe, i.e. ~2 Å [54]) and top facets tilted with respect to the substrates’ plane—thus suggesting that they are embedded in silver. Such Fe incorporation may appear surprising, taking into account the fact that iron generally does not alloy with silver. However, it has been shown that diffusion of iron into silver bulk is possible at high temperatures [55], therefore, we assume that at temperatures >500 K iron could be incorporated into the top silver layer. For higher iron coverages, mostly multilayer nanocrystals were observed (like the one marked with a green circle in the inset to Figure 3a), with shapes indicating <110>-oriented growth. Interestingly, the fraction of particles embedded in the substrate at the vicinity of the silver step edges was found to modify the steps direction (inset to Figure 3a, orange circle). In contrast to the RT deposition, no substrate etching was observed, which could be due to immediate surface smoothing at 550 K. The topography of the HT-FeO/Ag(111) sample also differed significantly from the RT-FeO/Ag(111): The HT film was inhomogeneous and consisted of different reconstructed and reconstruction-free surface species, most of which were located at the regular terrace sites, more uniformly distributed, had smaller sizes and less defined shapes.

Generally, the observed topographic features can be divided into the following categories:

(1) Islands exhibiting a 45 Å Moiré superstructure—virtually identical to the one observed on the RT-FeO/Ag(111) sample—some of which were growing on top of silver terraces and some were embedded into the top silver layer. An example of such an island is marked with a red arrow in Figure 3c;

(2) Strained embedded islands without Moiré. An example of such an island is marked with a blue arrow in Figure 3c. The atomically-resolved image, shown in Figure 3e, reveals an interatomic spacing of ~3.2 Å, which is very similar to the spacing observed on the Moiré-reconstructed islands. Strain-induced deformations, originating most probably from the embedment of the island into the top silver layer, are clearly visible on both STM images. Similar oxide island incorporation into the top silver layer was observed by other authors for MgO(001) and NiO(100) films on Ag(001) [56,57,58];

(3) Hexagonal islands with large in-plane atomic periodicity (~6 Å) and large heights (~5–15 Å). An example of such an island is marked with a green arrow in Figure 3c. The atomic periodicity, determined from the atomic-resolution STM image shown in Figure 3f, and height indicate that these islands represent the Fe_3_O_4_(111) phase [4];

(4) Structurally ill-defined islands—similar to those observed on the RT-FeO/Ag(111) sample;

(5) Atomically-flat islands with no visible signs of lattice mismatch-induced reconstructions. These islands were mostly growing at the lower silver step edges, occasionally being extended in between the particles growing at the regular terrace sites. Examples of such islands are marked with yellow arrows in Figure 3b), S1a and S1b. The height of the islands, ~2.4–2.5 Å, is very similar to the height of a monoatomic step on Ag(111) (~2.35 Å [54]), however, an atomically-resolved STM image, shown in Figure 3d), revealed that they consist of two superimposed atomic sublattices—a lattice of small, sharp protrusions (marked with black circles) and a lattice of a bigger and more diffuse-appearing species (yellow circles). A similarly spaced “lattice” of holes (blue circles) could be also observed. The contributions from different sublattices were visible on both topography and current images, however, with different intensities, therefore, the sum of the two images is presented (for separate topography and current images, see Figures S2a and S2b). The atomic periods within the respective lattices, determined from ~100 line profiles, were found to be 2.94 ± 0.08 Å. The standard deviation, obtained numerically, results from thermal drift and STM image distortion caused by room-temperature imaging at high scanning speed. Notably, the observed atomic structure was locally defect-free and exhibited no out-of-plane rumpling. Even though the height of these reconstruction-free islands and the interatomic distances within them were similar to those of clean Ag(111) (2.89 Å), the appearance of two atomic sublattices was somehow disturbing;

(6) Flat regions in between different types of islands. The height difference between such regions was ~2.35 Å and corresponded to the height of a monoatomic step on Ag(111). Interestingly, at higher oxide coverages (>1 ML), these regions were still present and became difficult to be scanned with STM using negative bias voltages (the tip was crashing the surface). This could indicate that these regions are not purely silver, but were structurally altered by iron deposition and oxidation.

We believe that the large variety of surface structures observed on the HT-FeO/Ag(111) sample compared to the RT-FeO/Ag(111) sample is related to the more uniform distribution of iron prior to oxidation and partial embedment of iron into the top silver layer.

**Figure 4:**
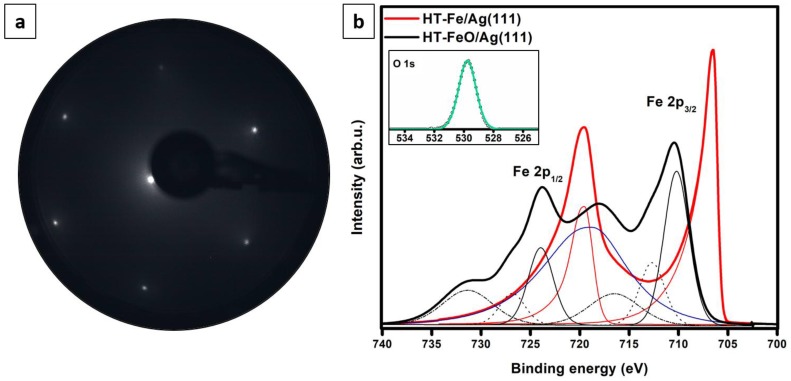
LEED pattern (60 eV) of 0.5 ML FeO film grown by Fe deposition onto Ag(111) substrate kept at 550 K and post-oxidation (**a**). (**b**) shows XPS Fe 2p spectra obtained for the 1.0 ML HT-Fe/Ag(111) (red line) and 1.0 ML HT-FeO/Ag(111) (black) samples. The Ag 3s signal is marked in blue. Inset in (b) presents the O 1s spectrum (green line) recorded for the oxidized sample.

The LEED pattern obtained for the oxidized sample shown in Figure 3b is presented in Figure 4a. Similarly to the RT-FeO/Ag(111) sample, the HT-FeO/Ag(111) sample exhibited a (1×1) LEED with no signatures of structural reconstructions. This may be related to the presence of different surface species, each of which contributed in an insignificant way to the observed pattern.

The XPS data recorded for the 1.0 ML HT-Fe/Ag(111) and 1.0 ML HT-FeO/Ag(111) samples are presented in Figure 4b. The Fe 2p signal obtained for the HT-Fe/Ag(111) sample could be fitted with two components, centered at around 706.4 eV and 719.6 eV, indicative of the presence of metallic iron. The spectrum obtained after oxidation had six contributions: 710.2 eV, 712.7 eV, 716.5 eV, 724.0 eV, 726.9 eV and 731.3 eV. The 710.2 eV and 724.0 eV peaks were assigned to originate from Fe^2+^ ions in iron oxide and the 712.7 eV and 726.9 eV components from multiplet splitting. The peaks at 716.5 eV and 731.3 eV were the characteristic Fe^2+^ satellite peaks. Similarly to the RT-FeO/Ag(111) sample, the binding energy difference of nearly 6 eV between the main Fe 2p_3/2_ photoemission line and the first satellite peak and nearly 7 eV between the main Fe 2p_1/2_ line and second satellite peak gave clear evidence for the dominant presence of the Fe^2+^ iron in the studied sample. Metallic iron, iron in the Fe^3+^ oxidation state (originating e.g. from the rarely observed Fe_3_O_4_ islands) or contaminants—if any—were beyond the detection limit of our XPS instrument. The oxygen O 1s signal recorded for this sample could be fitted with a single peak centered at around 529.8 eV. The Fe to O ratio determined from the survey spectrum (not shown) was approx. 1:1.

The obtained results reveal that slight modification of the oxide preparation procedure may lead to the formation of completely different surface structures: Deposition of Fe onto the Ag(111) substrate held at room temperature and post-oxidation in 1 × 10^−6^ mbar O_2_ at 700 K resulted in the formation of Moiré-reconstructed FeO islands located at the silver step edges, as well as ill-defined structures located at the regular terrace sites of silver, while deposition of iron onto a 550 K-heated substrate and post-oxidation resulted in a wide variety of uniformly distributed reconstructed and reconstruction-free surface species. Even though a slight change in the oxide growth conditions (iron amount, substrate temperature during deposition, oxygen pressure, oxidation temperature) was not critically affecting the structure of FeO films on other close-packed single-crystalline substrates used by different authors so far, the results obtained by us and other groups indicate that on Ag(111) these parameters play a crucial role. The co-existence of reconstructed and reconstruction-free islands with different atomic spacing following high-temperature iron deposition and post-oxidation, points to the high complexity of the Fe-O-Ag system and the need for further studies on this topic. These may be particularly significant with respect to the determination of the nature of the reconstruction-free islands. If, indeed, being Moiré-free FeO(111), such islands/films could constitute a perfect system for model-type electronic, catalytic and magnetic studies. The performed studies also reveal the necessity of using atomic-resolution techniques, such as STM, for the evaluation of the films structure, as XPS and LEED may provide misleading results.

In order to shed more light on the nature of the observed reconstruction-free islands, we performed point STS dI/dz measurements and compared the curves obtained on the islands with those recorded for clean Ag(111) and other single-crystalline substrate: Pt(111). The I(z) spectroscopy resembles the exponential change of the tunneling current with changing tip-sample distance [59]. The dependence can be measured even more precisely by recording the first derivative of the signal, i.e. dI/dz, using the lock-in technique [60,61]. As the slope of the dI/dz curve is proportional to the average work function of the sample and tip, the technique allows distinguishing surfaces with different work function values (assuming a constant work function of the tip during all the measurements). The I(z) method was, for example, successfully applied by other authors to determine local work function variations within the Moiré superstructure of FeO(111)/Pt(111) [62].

**Figure 5:**
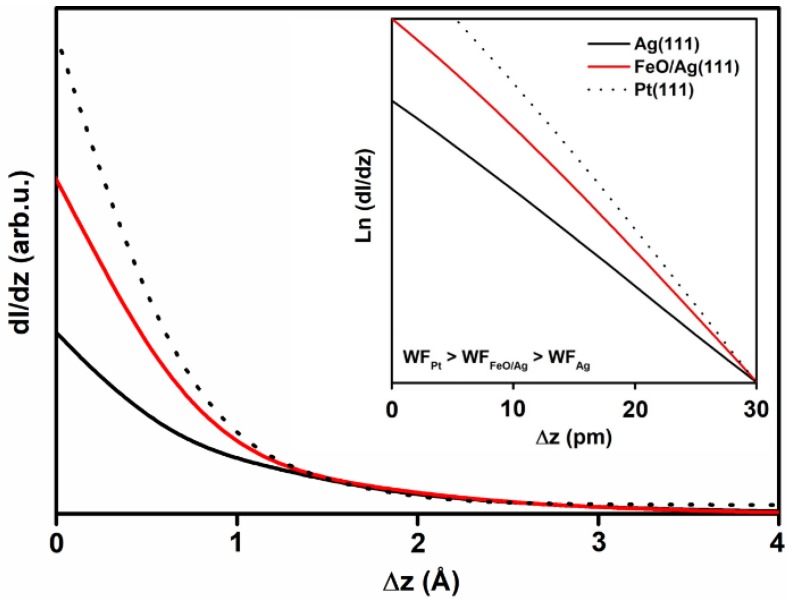
dI/dz curves obtained from clean Ag(111) (black solid curve), clean Pt(111) (black dotted curve) and the reconstruction-free islands grown by Fe deposition onto a Ag(111) substrate kept at 550 K and post-oxidation (red curve). The inset presents the slopes of the curves plotted in a logarithmic scale.

The recorded dI/dz curves are shown in Figure 5. The inset presents excerpts from the curves (slopes) plotted in a logarithmic scale. As expected, the slopes of the curves obtained for the probed single crystal surfaces differed significantly, in agreement with their different work function values (the literature values are 4.46–4.50 eV [36,37] and 6.08–6.4 eV [34,63,64,65] for Ag(111) and Pt(111), respectively). The slope of the curve obtained for the reconstruction-free islands on the HT-FeO/Ag(111) sample was much higher than that of clean Ag(111) and lower than that of Pt(111). The measurements were repeated several times for different sample preparations (and by changing all three samples in a random way to exclude changes in the tip condition in between the measurements) and the trend was always the same. Based on this, it may be concluded that the islands work function value is higher than the work function of silver and lower than the work function of platinum.

### 3.3. Calculations on a Conceptual Reconstruction-Free FeO/Ag(111)

In order to determine the structure and properties of a conceptual reconstruction-free FeO/Ag(111), the FeO film was modelled using a 1×1 surface unit cell, thus adopting the lattice constant of the Ag(111) support. This means that during the structural optimization of the systems, the FeO(111) lattice was slightly compressed to match the lattice of Ag(111). Such approach did not allow for any kind of in-plane reconstructions. The results were obtained for the calculated Ag bulk lattice constant of 4.155 Å (PBE) and FeO lattice constant of 4.288 Å (PBE+U). These values differ by 3.1%, while the experimentally-determined lattice parameters of Ag (4.086 Å) and FeO (4.32 Å) differ by 5.7%. Therefore, a similar set of calculations, including structural optimizations of the considered monolayer and bilayer FeO(111) systems, was performed using the experimental lattice constants of Ag and FeO. Importantly, the calculated geometries, presented in SM3, do not show substantial differences with respect to those obtained with theoretically determined lattice constant values.

**Figure 6:**
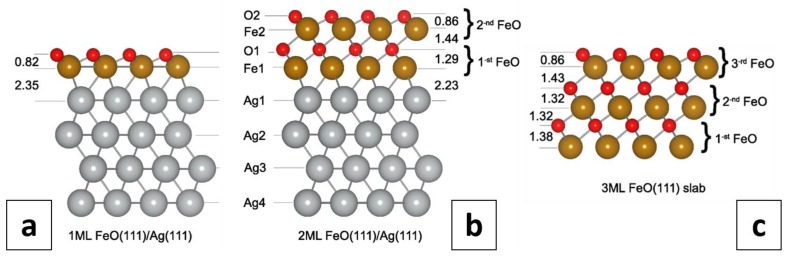
Side views of calculated O-terminated monolayer (**a**) and bilayer (**b**) reconstruction-free FeO(111) films on Ag(111), as well as 3-MLs-thick FeO slab (**c**), determined from PBE+U calculations using a 1×1 surface unit cell with fixed 2.938 Å FeO(111) and Ag(111) in-plane lattice constants (FeO(111) film adopting the geometry of the Ag(111) substrate). All distances are given in Å.

Several configurations of Fe-terminated and O-terminated FeO(111)/Ag(111) systems were examined, considering both Fe and O atoms placed in fcc or hcp hollows, as well as on top of Ag atoms. The most stable Fe-termination was found to be the one with Fe atoms occupying fcc positions and O atoms in the hcp hollow sites, with an O–Ag distance of 2.45 Å and an Fe–O distance of 0.90 Å. Other Fe-terminations were by at least 10 meV (with respect to the total energy) less favored. The most stable O-termination was again the one with Fe and O atoms sitting in the threefold coordinated fcc and hcp hollow sites of the Ag(111) surface, respectively, with an Fe–Ag distance of 2.35 Å and an O–Fe distance of 0.82 Å. This configuration (presented in Figure 6a) was by 0.79 eV energetically more favored than the Fe-terminated one. It has to be mentioned that for the 1×1 supercell calculations, this configuration was nearly degenerated in energy with the one with Fe in hcp and O in fcc sites (which was less stable by only 2 meV). The calculations performed using a 2×2 supercell confirmed the same preference of adsorption sites with a total energy difference of 6 meV, and those performed using the experimental lattice constant of Ag (4.086 Å) showed that the two configurations differ by 15 meV. Another O-termination, with O atoms in the on-top positions and Fe atoms in either fcc or hcp sites, were by 89 meV less favored. Based on those findings, further calculations were performed for the most energetically-stable O-terminated structure with Fe in the threefold coordinated fcc sites and O in the hcp hollow sites.

The calculated total height of the O-terminated monolayer FeO(111) film was found to be 3.17 Å. A strongly reduced Fe–O interlayer spacing (0.82 Å) in the FeO(111)/Ag(111) system compared to bulk FeO (1.25 Å) [66] is of similar magnitude to that reported for FeO(111)/Pt(111) [66] and results from a complex stabilization mechanism, i.e. an interplay between structural relaxation, polarity compensation, charge transfer and magnetic ordering [67,68,69,70]. The calculated adhesion energy of the FeO(111) monolayer on Ag(111) was found to be 0.91 eV per Fe atom. This is much less than that reported for the FeO(111) monolayer Pt(111) (1.36 eV [18]), but it is doubled compared to the value obtained for FeO(111) on Ag(100) (0.46 eV [18]). The difference in the adhesive bond strength is reflected in the structural properties of the FeO film. The Fe–Ag distance for FeO(111) supported on Ag(111) and Ag(100) is, respectively, 2.35 Å and 2.65 Å, whereas the Fe–Pt distance at the FeO/Pt(111) interface is ~2.7 Å (it varies slightly over different high-symmetry regions of the Moiré supercell) [31]. Interestingly, the reduction of the FeO-substrate bonding distance with the enhanced adhesive energy strength does not lead to a regular change in the distance between oxygen and iron planes in the FeO(111) monolayer (rumpling), which is largest (0.82 Å, Figure 6a) for FeO/Ag(111) and smallest (0.26 Å [18]) for FeO/Ag(100). For the FeO/Pt(111) it is ~0.7 Å [31]. The difference between the values obtained for FeO(111)/Ag(111) (this work) and FeO(111)/Ag(100) [18] may originate from different substrate orientation (Ag(111) vs. Ag(100)) and the computational unit cell used (1×1 vs. p(2×11)) which, in our case, did not allow for any in-plane relaxations. An inspection of the calculated Bader charges (SM4 and Figure S3) on the interface atoms of the FeO(111) monolayer deposited on a Ag(111) substrate showed that adsorption of a FeO film results in the appearance of negative charges on silver (0.17 |e|) and iron (0.04 |e|) atoms, gained at the expense of oxygen (0.24 |e|). This charge transfer may be important with respect to films stabilization. Another stabilizing factor may be the magnetic superstructure. It is worth noting that the applied 1×1 (or a larger 2×2) surface unit cell gives a ferromagnetic (FM) ground state and does not allow an antiferromagnetic (AFM) superstructure, which results from magnetic frustration of antiferromagnetic FeO on the three-fold symmetric oxide layer, to be reproduced [31]. Such a magnetic superstructure is observed, indeed, if a 3×3 or a ($\sqrt{3}$×$\sqrt{3}$)R30° surface unit cell is applied, where the number of Fe atoms in a layer is a multiple of three with a 1:2 ratio of Fe atoms with opposite magnetic moments. In that case, the AFM structure is by 0.26 eV per FeO unit more stable than the FM one. However, the AFM alignment of magnetic moments on Fe atoms makes the iron layer distinctly rumpled, with Fe atoms of one direction of magnetization being by around 0.5 Å more distant from the Ag(111) surface than those with opposite magnetization (Figure S4). Such rumpling could be, potentially, experimentally observed below the Néel temperature of FeO (~198 K [71]).

The bilayer FeO(111) film was modelled by placing a second monolayer of FeO(111) on top of the FeO(111)/Ag(111) slab, originally in a flat configuration, with Fe over surface Ag atoms and O atoms in fcc positions over Fe atoms in the first FeO layer (Figure 6b). Upon structural optimization, the lateral positions of Fe and O atoms remained unchanged and the atoms relaxed only vertically. Both FM and AFM stackings of ferromagnetically ordered adjacent FeO layers were considered. The AFM configuration appeared to be by 63 meV more stable than the FM one and, thus, it was adopted in further calculations. The height of the first (interface) monolayer increased to 3.52 Å due to a substantial increase of the O–Fe distance (to 1.29 Å). The thickness of the second (top) monolayer was 2.30 Å, with an O–Fe distance of 0.86 Å. The distance between the iron oxide layers was 1.44 Å and the distance between the Fe atoms in the bottom layer and the silver support was reduced to 2.23 Å. These values suggest strengthening of the adhesive bond between the bilayer FeO(111) and the silver support compared to monolayer FeO film on Ag(111). It has to be mentioned that the results obtained for monolayer and bilayer FeO(111) films did not change with a change of the supercell from 1×1 to 2×2. Notably, the height of the experimentally observed reconstruction-free islands (2.4–2.5 Å) differs significantly from the value calculated for a monolayer FeO on Ag(111) (3.2 Å). In fact, the experimental value is more similar to the height of the calculated second FeO layer on Ag(111) (2.3 Å). It is, of course, possible that the height of the experimentally observed structures, if indeed being FeO, is affected by stabilization mechanisms that were not taken into account in our theoretical considerations. One could also speculate that the experimentally observed structures are the second-layer FeO islands which grow on top of the first-layer FeO islands embedded in the silver substrate. This would make FeO/Ag(111) system similar to MgO(001)/Ag(001) and NiO(100)/Ag(001) [56,57,58]. On the other hand, the second FeO(111) layer on Pt(111) was reported to undergo strong structural relaxations resulting from polarity compensation [72,73].

**Figure 7:**
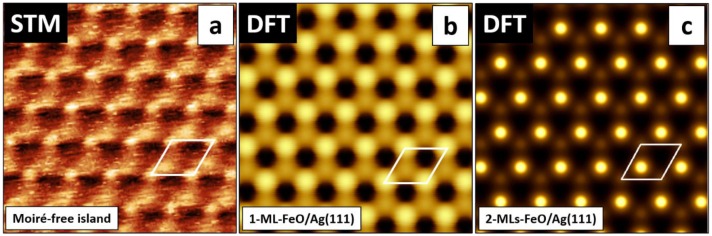
Atomic-resolution STM image (1.73 × 1.73 nm^2^, V = +0.1 V, I = 0.4 nA) obtained on the reconstruction-free island on the HT-FeO/Ag(111) sample (**a**). (**b**) and (**c**) present simulated images of a 1-ML-thick and 2-MLs-thick reconstruction-free FeO(111) films on Ag(111), respectively (see text for details). The simulation is based on density functional theory (DFT) calculations and the Tersoff-Hamann method [41]. The FeO(111)-(1×1) unit cell is marked with a white rhombus.

To compare the atomic structures, we simulated, based on the calculated geometries, the STM images of 1 ML FeO/Ag(111) and 2 MLs FeO/Ag(111) and set them side by side with the atomic-resolution image obtained experimentally on one of the reconstruction-free islands (Figure 7a–c, respectively). The simulated STM images for 1-ML-thick and 2-MLs-thick films differ by contrast, however, they both seem to fit the experimental image, where the smaller protrusions observed experimentally would correspond to iron atoms, the bigger to oxygen atoms and the holes to the positions of silver atoms.

In order to check the influence of the Ag(111) substrate on the structure of 1-ML-thick and 2-MLs-thick FeO(111) films, we also simulated a free-standing 3-MLs-thick (six atomic layers) FeO(111) slab and compared its parameters with those of Ag(111)-supported films (see Figure 6c and SM4). As the antiferromagnetic configuration of alternating layers was found to be by 2.01 eV more favored than the ferromagnetic one, the further description is given for the AFM phase. The slab is asymmetric due to differently terminated sides which makes the interlayer separations at the O-terminated and Fe-terminated sides, respectively, shrink or expand. Notably, the distances between the inner Fe and O planes (1.32–1.43 Å) were found to be close to the calculated Fe–O spacing in a bilayer FeO(111) on Ag(111) (1.29–1.44 Å) and in bulk FeO (1.25 Å) [66]. The similarity between the Ag(111)-supported bilayer FeO(111) film and a free-standing 3-MLs-thick FeO(111) slab was also reflected in the calculated Bader charges and magnetic moments (SM4). The magnitude of both electron charges and magnetic moments on atoms of the top surface layer of a bilayer film and a free-standing slab is mainly determined by the presence of the surface. In both structures, the O and Fe atoms of the outermost layer lose 0.25 and 0.41–0.43 electrons, respectively. The electron charge gain on Fe1 atoms of the bilayer (0.51 |e|) and Ag1 atoms of the substrate (0.19 |e|) indicate the weak character of the FeO–Ag bonding. The magnetic moments on atoms of the outermost FeO layers of the bilayer and a free-standing 3-MLs-thick slab are nearly the same, which means that they are determined by the presence of both the surface and the underlying FeO monolayer. In contrast, when a monolayer FeO is placed directly on Ag(111), magnetic moments on oxygen atoms (0.39 μB) are induced and the moments on Fe atoms are enhanced compared to “FeO-supported” FeO layer.

**Figure 8:**
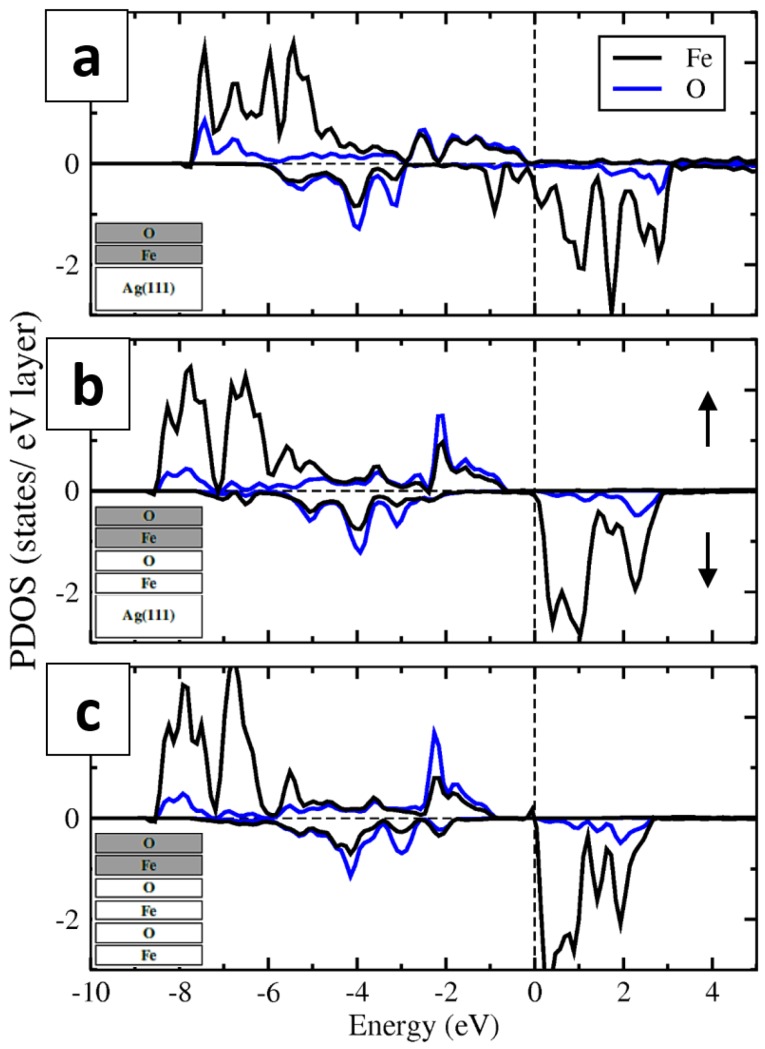
Partial densities of electronic states (PDOS) of a FeO(111) monolayer on Ag(111) (**a**), second FeO(111) layer from a bilayer FeO(111) film on Ag(111) (**b**) and the top FeO layer from a free-standing 3-MLs-thick FeO(111) slab (**c**), resulting from PBE+U calculations.

The partial densities of electronic states (PDOS) calculated for the FeO(111) monolayer on Ag(111), the second FeO layer from a bilayer FeO(111) film on Ag(111) and the top FeO layer from the 3-MLs-thick FeO slab are presented in Figure 8a–c. The similarity between the electronic structure of all three systems can be seen, especially when comparing the second FeO layer on Ag(111) and a free-standing FeO slab. It suggests that the density of states is mainly determined by the presence of the surface and not by the interaction with the Ag(111) support. However, the presence of the underlying FeO layer is important for the opening of an energy gap of 0.7–0.9 eV in the topmost FeO layer (in the majority spin states; see Figure 8b,c). The Fe-DOS is dominated by the Fe 3d states and the O-DOS by the O 2p states. There is also a great asymmetry in the majority and minority spin states, in particular in those belonging to Fe. The former is almost completely occupied, whereas the latter are mostly empty.

Based on the performed calculations, it may be concluded that the second FeO(111) layer on Ag(111) has a “free-standing FeO” character.

The calculated work function values of a monolayer FeO(111) film on Ag(111) range from 5.87 eV (AFM configuration, ($\sqrt{3}$×$\sqrt{3}$)R30° unit cell) to 6.56 eV (FM configuration, 1×1 unit cell) (see SM5 for values obtained for different configurations of magnetic arrangements and computational unit cells). Bilayer FeO has an even higher work function value: 6.67 eV. Notably, both values are much higher than the work function of clean Ag(111). The value roughly determined by us experimentally using dI/dz spectroscopy for the reconstruction-free islands seems to be similar to that calculated for monolayer FeO/Ag(111) and lower than the one calculated for bilayer FeO.

## 4. Conclusions

We used STM, LEED and XPS to study ultrathin FeO films grown on Ag(111) using two different procedures: (1) By depositing iron onto a silver substrate kept at room temperature and post-oxidizing it and (2) by depositing iron onto a silver substrate kept at 550 K and post-oxidizing. Following the procedure (1), we observed the formation of islands exhibiting a 45 Å Moiré superstructure. These islands grew mainly at the silver step edges, which is related to iron agglomeration at these surface sites following room-temperature deposition. Procedure (2) was found to lead to the formation of various reconstructed and reconstruction-free surface species, where the reconstruction-free ones grew mainly at the lower silver step edges, while the other species were uniformly distributed across the silver surface. The difference in the growth mode compared to procedure (1) is related to the formation of uniformly distributed bcc Fe(110) crystallites following 550 K deposition and partial embedment of iron/iron oxide in the top silver layer. The results of this and other works on the FeO/Ag(111) indicate the critical role of the growth conditions on the structure of the obtained films. To the best of our knowledge, such dependence has not been observed for FeO on any other close-packed single-crystalline substrate so far. It is now evident that at least two different Moiré superstructures may be formed for FeO/Ag(111), i.e. the 26 Å 8 FeO×9 Ag superstructure ([17,18,19]) and the 45 Å 14 FeO×9$\sqrt{3}$ Ag (this work), the growth of which is accompanied by the formation of various ill-defined and reconstruction-free surface species. With respect to the reconstruction-free species, their unambiguous compositional identification is not trivial, however, their atomic structure and electronic characteristics seem to differ from those of clean Ag(111), thus it is tempting to assign them to Moiré-free FeO. The performed DFT calculations indicate that a reconstruction-free FeO(111) film on Ag(111), if successfully grown, should exhibit tunable thickness-dependent properties, being substrate-influenced in the monolayer regime and free-standing-FeO-like when in the bilayer form. The latter could constitute a perfect model system for electronic, catalytic and magnetic studies.

## Supplementary Materials

The following are available online at http://www.mdpi.com/2079-4991/8/10/828/s1, Overview of the literature data concerning the preparation of iron oxide films on silver single crystal supports, detailed X-ray photoelectron spectroscopy data, geometrical details of the calculated FeO systems, numerically-determined Bader charges, magnetic moments and work function values, additional figures.
