# Supplementary material for: On the Structure of Ultrathin FeO Films on Ag(111)
Source: Nanomaterials (Basel). 2018 Oct 13;8(10):828. doi: 10.3390/nano8100828 (PMC6215274; doi:10.3390/nano8100828)
Supplement: Supplementary file 1 [file nanomaterials-08-00828-s001.pdf]

Supplementary Materials

SM1. Overview of the literature data concerning the preparation of iron oxide films on silver single crystal supports (chronological order).

Ref.	Iron Oxide	Support	Preparation Procedure	Authors' Description	Methods
[1]	FeO(111), Fe_3O_4(111)	Ag(111)	Repeated cycles of submonolayer iron deposition and oxidation: FeO(111) for initial 3–4 MLs, Fe_3O_4(111) for thicker films	No detailed description	LEED, XPS, XPD
[2]	FeO(111) (<6 ML), Fe_3O_4(111) (>6 ML) (also denoted as "FeO_x")	Ag(111)	1–10 MLs Fe at RT<br>10^{-5} Torr O_2 at 350 °C for 15 min<br>Cooled down in O_2 to 200 °C<br>UHV annealed at 400 °C for 30 min	Poorly ordered FeO(111), broadening of LEED spots and background increase, XPS indicated mixed FeO and Fe_3O_4 oxide phases or non-stoichiometric oxides	LEED, XPS, XPD
[2]	FeO(111) (<10 Å), Fe_3O_4(111) (>10 Å) (also denoted as "FeO_x")	Ag(111)	Multiple cycles ≤0.5 ML Fe at RT<br>Heat to 150 °C (prior to O_2 introduction)<br>10^{-5} Torr O_2 at 150 °C for 5 min<br>UHV annealed at 400 °C for 30 min	Good crystalline quality FeO(111), "split" LEED beams for FeO(111), (2×2) LEED for Fe_3O_4, for thicker films XPS indicated mixed FeO and Fe_3O_4 oxide phases	LEED, XPS, XPD
[3]	FeO(001)	Ag(001)	22 MLs ^{57}Fe at RT in 1 × 10^{-7} mbar O_2<br>UHV annealed at 600 °C for 10 min	Stoichiometric FeO(001) with good crystalline quality, well defined LEED, only Fe^{2+} in XPS, ~15% of Fe_3O_4 underneath FeO	LEED, Mössbauer
[4]	FeO(001), FeO(111)	Ag(001)	Not mentioned	Nearly bulk terminated FeO(001) and FeO(111)	LEED
[5]	FeO(111) (<10 Å), Fe_3O_4(111) (>10 Å), Fe_2O_3(0001)	Ag(111)	Multiple cycles of Fe deposition (amount not indicated) at	FeO(111) with lattice constant close to bulk	LEED, XRD, Raman

	(from $Fe_3O_4(111)$)		temperatures from RT to 150 °C<br>Oxidation:<br>2×10^{-6} mbar O_2 at temperatures up to 400 °C (FeO(111))<br>5×10^{-6} mbar O_2 at 150 °C for 15 min ($Fe_3O_4(111)$),<br>UHV annealed at >350 °C<br>9×10^{-6} mbar O_2 at 450 °C (flash) ($Fe_3O_4(111)$ → $Fe_2O_3(0001)$)	value, well ordered epitaxial $Fe_3O_4(111)$ and $Fe_2O_3(0001)$ films in two domains	
[6]	FeO(111) (<10 Å), $Fe_3O_4(111)$ (>10 Å), $Fe_2O_3(0001)$ (from $Fe_3O_4(111)$)	Ag(111)	Same as in Ref. [4]	This article is an extended version of Ref. [4], phase transformations between Fe_2O_3 and Fe_3O_4 possible	SXRD, LEED, Raman
[7]	FeO(001), FeO(111), $Fe_3O_4(001)$	Ag(001)	Fe deposition at RT<br>10^{-5} mbar O_2 at 300 °C for 1h	FeO(001) wetting the surface, $Fe_3O_4(001)$-like structure, quasi-hexagonal FeO(111)	LEED, STM, XPS
[7]	FeO(111)	Ag(001)	Fe deposition at 200–400 °C in 10^{-5} mbar O_2	Quasi-hexagonal FeO(111)	LEED, SPA-LEED, XPS, AES, STM
[8]	FeO(100), Fe_3O_4	Ag(100)	4–80 MLs ^{57}Fe deposition at RT (or higher) in 5.0×10^{-8} mbar to 4.6×10^{-7} mbar O_2<br>UHV annealed at 420–660 °C for 1–7 min	Well defined FeO/Fe_3O_4 samples with different phase content ratio	XPS, Mössbauer, LEED
[9]	FeO(111)	Ag(001)	0.5 ML at 570 K in 10^{-5} mbar O_2	Quasi-hexagonal FeO(111)	XPS, STM, LEED
[10]	FeO(111), mixed FeO(111)/FeO(100), Fe_2O_3	Ag(100)	Fe deposition at 25–400 °C in 2×10^{-7} mbar, 1×10^{-6} mbar and 5×10^{-6} mbar O_2	ML FeO(111) with FeO(100)-like grain boundaries, mixed FeO(111)/FeO(100) structure, hexagonal multilayer with buckled top layer—attributed to Fe_2O_3	STM, LEED, XPS, NEXAFS
[11]	α-$Fe_2O_3(0001)$, $Fe_3O_4(111)$	Ag(111)	Cycles of 7–9 MLs Fe deposition at RT	Well-ordered films, phase	LEEM, LEED

			2×10^{-6} mbar O_2 at 720 K (Fe_3O_4) 2.4×10^{-5} mbar at 670 K (Fe_2O_3)	transformations possible	
[12]	FeO(111), FeO(100) (also denoted as "FeO_x")	Ag(100)	0.4-2.0 MLs at 100 °C in 1×10^{-5} mbar to 2×10^{-7} mbar O_2 UHV annealed at 400 °C for 2 min	p(2×11) / c(2×12) unit cell coincidence structures, multilayer FeO	STM, LEED, XPS, NEXAFS
[13]	α-Fe_2O_3(0001), γ-Fe_2O_3(111), Fe_3O_4(111)	Ag(111)	Same as in Ref. [10], 3×10^{-5} mbar at <620 K for 10 min (γ-Fe_2O_3(111))	Well-ordered films, transformations between phases possible	LEEM, LEED, XPEEM
**[Error! Reference source not found.]**	Fe_3O_4(111)	Ag(001)	Fe deposition at RT in 2×10^{-6} mbar O_2 UHV annealed at 675 K	Fe_3O_4(111) growing in two crystallographic domains	LEED, SXRD, STM
[14]	Fe_3O_4(001)	Ag(001)	Fe deposition at RT ~10^{-6} mbar O_2 at RT for 10 min ~2×10^{-7} mbar O_2 at 650 K for 30 min	Fe_3O_4(001) with a $(\sqrt{2}\times\sqrt{2})$R45° reconstruction	LEED, SXRD, STM
[14]	Fe_3O_4(001)	Ag(001)	Fe deposition at RT ~3×10^{-7} mbar O_2 at RT for 10 min ~3×10^{-7} mbar O_2 at 650 K for 30 min and 770 K for 30 min	Seed layer for the growth of a well-ordered (001) magnetite layer	LEED, SXRD, STM
[15]	FeO(111), Fe_3O_4(111), multilayer FeO_x	Ag(111)	Fe deposition at various substrate temperatures (RT to 773 K) in 2×10^{-7} Torr to 1×10^{-6} Torr O_2	FeO(111) with a p(9×9) Moiré superstructure, Fe_3O_4(111), FeO(111)-like multilayer structure denoted as "FeO_x"	LEED, TPD, RAIRS
[16]	FeO(111)	Ag(100)	Same as in Ref. [11]	Same as in Ref. [11]	STM, LEED, RAIRS, TPD, DFT+U
[17]	FeO(111)	Ag(100), Ag(111)	Fe deposition at 100 °C in 2×10^{-7} mbar O_2 UHV annealed at 400 °C	Monolayer FeO(111) with coincidence structures same as in Ref. [15]	STM, LEED, SXRD, TPD, RAIRS

This work	FeO(111)	Ag(111)	1 ML Fe at RT<br>1 × 10^{-6} mbar O_2 at 700 K for 30 min	30°-rotated 14×14 FeO on 9√3×9√3 Ag Moiré superstructure and various ill-defined structures	STM, LEED, XPS
This work	FeO(111), Fe_3O_4(111)	Ag(111)	1 ML Fe at 500–600 K<br>1 × 10^{-6} mbar O_2 at 700 K for 30 min	Moiré-reconstructed and reconstruction-free, stoichiometric and well-ordered FeO(111)	STM, LEED, XPS

"ML" indicates "monolayers", "RT"—"room temperature", "UHV"—ultra-high vacuum. The scientific units are the same as in the original articles.

References

1. Waddill, G.D.; Ozturk, O. Growth of Ultrathin Iron Oxide and Chromium Oxide Films on Ag(001) and Ag(111). In Proceedings of the Annual American Physical Society March Meeting, Indiana Convention Center, Indianapolis, March 18-22, 2002; #H33.095.
2. Waddill, G.D.; Ozturk, O. Epitaxial growth of iron oxide films on Ag(111). *Surf. Sci.* **2005**, *575*, 35–50.
3. Lopes, E.L.; Abreu, G.J.P.; Paniago, R.; Soares, E.A.; de Carvalho, V.E.; Pfannes, H.-D. Atomic geometry determination of FeO(001) grown on Ag(001) by low energy electron diffraction. *Surf. Sci.* **2007**, *601*, 1239–1245.
4. Lopes, E.L.; Abreu, G.J.P.; Paniago, R.; Soares, E.A.; Pfannes, H.-D.; de Carvalho, V.E. Low energy electron diffraction analysis of the (001) and (111) surface geometry of FeO films grown on Ag(001) surface. *J. Electr. Spectr. Rel. Phenom.* 2007, 156–158.
5. Schlueter, C.; Lübbe, M.; Gigler, A.M.; Moritz, W. Growth of Iron-Oxides on Ag(111) Surfaces. *Macla* **2010**, *13*, 203–204.
6. Schlueter, C.; Lübbe, M.; Gigler, A.M.; Moritz, W. Growth of iron oxides on Ag(111)—reversible Fe_2O_3/Fe_3O_4 transformation. *Surf. Sci.* **2011**, *605*, 1986–1993.
7. Bruns, D. Structure and morphology of ultrathin iron and iron oxide films on Ag(001). Ph.D. Thesis, Universität Osnabrück, Germany, 2012.
8. Abreu, G.J.P.; Paniago, R.; Pfannes, H.-D. Growth of ultra-thin FeO(100) films on Ag(100): A combined XPS, LEED and CEMS study. *J. Magn. Magn. Mater.* **2014**, *349*, 235–239.
9. Bruns, D.; Kiesel, I.; Jentsch, S.; Lindemann, S.; Otte, C.; Schemme, T.; Kuschel, T.; Wollschläger, J. Structural analysis of FeO(111)/Ag(001): Undulation of hexagonal oxide monolayers due to square lattice metal substrates. *J. Phys.: Condens. Matter* **2014**, *26*, 315001.
10. Ataran, S. Iron Oxide Thin Film Growth On Ag(100). M.Sc. Thesis, Lund University, Sweden, 2014.
11. Genuzio, F.; Sala, A.; Schmidt, Th.; Menzel, D.; Freund, H.-J. Interconversion of α-Fe_2O_3 and Fe_3O_4 Thin Films: Mechanisms, Morphology, and Evidence for Unexpected Substrate Participation. *J. Phys. Chem. C* **2014**, *118*, 29068–29076.
12. Merte, L.R.; Shipilin, M.; Ataran, S.; Blomberg, S.; Zhang, C.; Mikkelsen, A.; Gustafson, J.; Lundgren, E. Growth of Ultrathin Iron Oxide Films on Ag(100). *J. Phys. Chem. C* **2015**, *119*, 2572–2582.
13. Genuzio, F.; Sala, A.; Schmidt, Th.; Menzel, D.; Freund, H.-J. Phase transformations in thin iron oxide films: Spectromicroscopic study of velocity and shape of the reaction fronts. *Surf. Sci.* **2016**, *648*, 177–187.
14. Lamirand, A.D.; Grenier, S.; Langlais, V.; Ramos, A.Y.; Tolentino, H.C.N.; Torelles, X.; De Santis, M. Magnetite epitaxial growth on Ag(001): Selected orientation, seed layer, and interface sharpnes. *Surf. Sci.* **2016**, *647*, 33–38.
15. Mehar, V.; Merte, L.R.; Choi, J.; Shipilin, M.; Lundgren, E.; Weaver, J.F. Adsorption of NO on FeOx Films Grown on Ag(111). *J. Phys. Chem. C* **2016**, *120*, 9282–9291.

16. Merte, L.R.; Heard, C.J.; Zhang, F.; Choi, J.; Shipilin, M.; Gustafson, J.; Weaver, J.F.; Grönbeck, H.; Lundgren, E. Tuning the Reactivity of Ultrathin Oxides: NO Adsorption on Monolayer FeO(111). *Angew. Chem. Int. Ed.* **2016**, *55*, 9267–9271.
17. Shipilin, M.; Lundgren, E.; Gustafson, J.; Zhang, C.; Bertram, F.; Nicklin, C.; Heard, C. J.; Grönbeck, H.; Zhang, F.; Choi, J.; et al. Fe Oxides on Ag Surfaces: Structure and Reactivity. *Top. Catal.* **2017**, *60*, 492–502.

SM2. Detailed X-ray photoelectron spectroscopy data.

	RT-Fe/Ag(111)		HT-Fe/Ag(111)	
**Component**	Fe $2p_{3/2}$	Fe $2p_{1/2}$	Fe $2p_{3/2}$	Fe $2p_{1/2}$
**Position (eV)**	706.6	719.9	706.4	719.6
**FWMH**	0.7	2.0	0.87	2.0
**Area**	181133.3	90566.6	95668.4	47834.2

	RT-FeO/Ag(111)						
	Fe $2p_{3/2}$			Fe $2p_{1/2}$			O 1s
**Component**	Fe^{2+}	Multiplet splitting	Satellite	Fe^{2+}	Multiplet splitting	Satellite	$O^{(2-)}$
**Position (eV)**	710.3	712.6	716.6	724.0	726.9	731.6	529.7
**FWMH**	3.0	3.0	0.87	3.0	3.0	5.5	1.4
**Area**	81849.0	40999.9	26261.2	40924.5	20500	28222.0	79223.4
**Concentration (%)**	53.5%						46.5%

	HT-FeO/Ag(111)						
	Fe $2p_{3/2}$			Fe $2p_{1/2}$			O 1s
**Component**	Fe^{2+}	Multiplet splitting	Satellite	Fe^{2+}	Multiplet splitting	Satellite	$O^{(2-)}$
**Position (eV)**	710.2	712.7	716.5	724.0	726.9	731.3	529.8
**FWMH**	3.0	3.0	6.0	3.0	3.0	6.0	1.4
**Area**	63063.1	26488.7	26113.4	31531.6	13244.4	28222.0	55929.4
**Concentration (%)**	54.7%						45.3%

The fittings confirmed that after the oxidation the iron is in the Fe^{2+} oxidation state. The presence of significant amounts of other Fe^{2+} containing iron oxide phase—Fe_3O_4, can be excluded, as it consists of a mixture of Fe^{2+} and Fe^{3+} ions [18,19]. Fe_2O_3, on the other hand, consists of iron in the Fe^{3+} oxidation state only.

References

18. Hawn, D.D.; DeKoven, B.M. Deconvolution as a correction for photoelectron inelastic energy losses in the core level XPS spectra of iron oxides. *Surf. Interface Anal.* **1987**, *10*, 63–74.
19. Muhler, M.; Schlögl, R.; Ertl, G. The nature of the iron oxide-based catalyst for dehydrogenation of ethylbenzene to styrene 2. Surface chemistry of the active phase. *J. Catal.* **1992**, *138*, 413–444.

SM3. Geometrical details of the calculated FeO systems.

Separations of atomic layers along the coordinate perpendicular to the (111) surface plane calculated for the monolayer FeO(111) film on Ag(111), the bilayer FeO(111) on Ag(111) and a free-standing 3-monolayers-thick FeO slab, obtained from GGA+U calculations using 1×1 surface unit cells. The numbering of atomic layers is referenced to the topmost Ag layer. The numbers in brackets refer to the geometrical parameters resulting from structural optimization of the systems constructed using the experimental lattice constant of Ag (round brackets) and PBE+U lattice constant of FeO [square brackets]. In a free-standing 3 MLs FeO slab, the layers are numbered from the top (terminating O layer) to the bottom. In bulk FeO, the in-plane interatomic Fe–Fe and O–O distances are 3.04 Å and the separation of Fe and O planes is 1.25 Å [20]. In the performed calculations, the calculated in-plane lattice constant of FeO is equal to the surface lattice constant of Ag(111) due to the use of a 1×1 computational cell. ~5% contraction of the surface lattice constant with respect to bulk FeO is of much smaller magnitude than those reported for FeO(111) films on other single-crystalline substrates.

System	Layers	Separation (all values in Å)
**Monolayer FeO(111)/Ag(111)**	O1-Fe1	0.82 (0.86)
**Monolayer FeO(111)/Ag(111)**	Fe1-Ag1	2.35 (2.36)
**Bilayer FeO(111)/Ag(111)**	O2-Fe2	0.86 (0.89)
**Bilayer FeO(111)/Ag(111)**	Fe2-O1	1.44 (1.44)
**Bilayer FeO(111)/Ag(111)**	O1-Fe1	1.29 (1.33)
**Bilayer FeO(111)/Ag(111)**	Fe1-Ag1	2.23 (2.26)
**3-MLs-FeO slab**	O1-Fe1	0.86 [0.79]
**3-MLs-FeO slab**	Fe1-O2	1.43 [1.41]
**FeO(111) surface**	In-plane lattice constant	PBE: 2.938 (fixed)<br>[PBE+U: 3.032] (fixed)

References

20. Kim, Y.J.; Westphal, C.; Ynzunza, R.X.; Galloway, H.C.; Salmeron, M.; van Hove, M.A.; Fadley, C.S. Interlayer interactions in epitaxial oxide growth: FeO on Pt(111). *Phys. Rev. B* **1997**, *55*, R13448–R13451.

SM4. Calculated Bader charges and magnetic moment on atoms.

Bader charges and magnetic moments on atoms within different layers. The charge on atom is calculated as the difference between the calculated Bader charge on atom of a given layer minus the charge on respective Fe (6.55 |e|) or O (7.45 |e|) atom in bulk FeO and on Ag1 atom of the clean Ag(111) slab (11.02 |e|), respectively. The magnetic moment on individual Fe atoms of bulk FeO is ±3.65 μ_B and zero on O atoms. The labeling of the layers refers to that shown in Figure 6 in the main text. The entries in the column labeled as "3 ML slab" refer to the free-standing 3-MLs-thick FeO(111) slab, calculated using the PBE lattice constant of Ag and the PBE+U lattice constant of FeO.

Layer	Bader Charge (in |e|)			Magnetic Moment (μ_B)		
	1 ML	2 ML	3 ML slab	1 ML	2 ML	3 ML slab
O3			−0.25 (−0.23)			0.46 (0.44)
Fe3			−0.43 (−0.44)			4.16 (4.18)
O2		−0.25	−0.04 (−0.06)		0.45	0.07 (0.09)
Fe2		−0.41	−0.02 (0.00)		4.15	−3.60 (−3.58)
O1	−0.24	−0.07	−0.03 (−0.05)	0.39	0.05	0.00 (0.01)
Fe1	0.04	0.51	0.76 (0.78)	3.85	−3.41	3.48 (3.48)
Ag1	0.17	0.19		0.00	0.02	

SM5. Calculated work function values.

Calculated Work Function Values (eV)	
Ag(111) (1×1)	4.49
1ML FeO/Ag(111)-FM (1×1)	6.56
1ML FeO/Ag(111)-FM (√3×√3)	6.57
1ML FeO/Ag(111)-AFM (same result for 1×1 and √3×√3)	5.87
2ML FeO/Ag(111)-AFM (1×1)	6.67
2ML FeO/Ag(111)-AFM (√3×√3)	6.69
3ML FeO slab-AFM (same result for 1×1 and √3×√3)	6.95

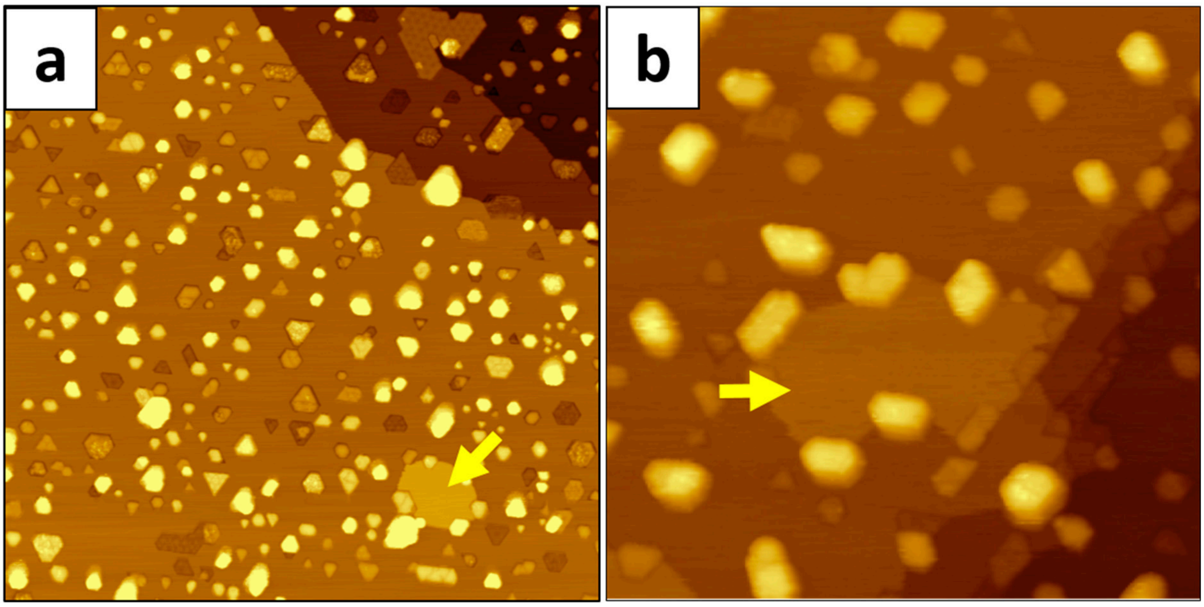

Figure S1. STM images of reconstruction-free islands (marked with yellow arrows) observed on different HT-FeO/Ag(111) samples (300 × 300 nm^2, V = +1.0 V, I = 0.4 nA (**a**) and 50 × 50 nm^2, V = +0.7 V, I = 0.4 nA (**b**)).

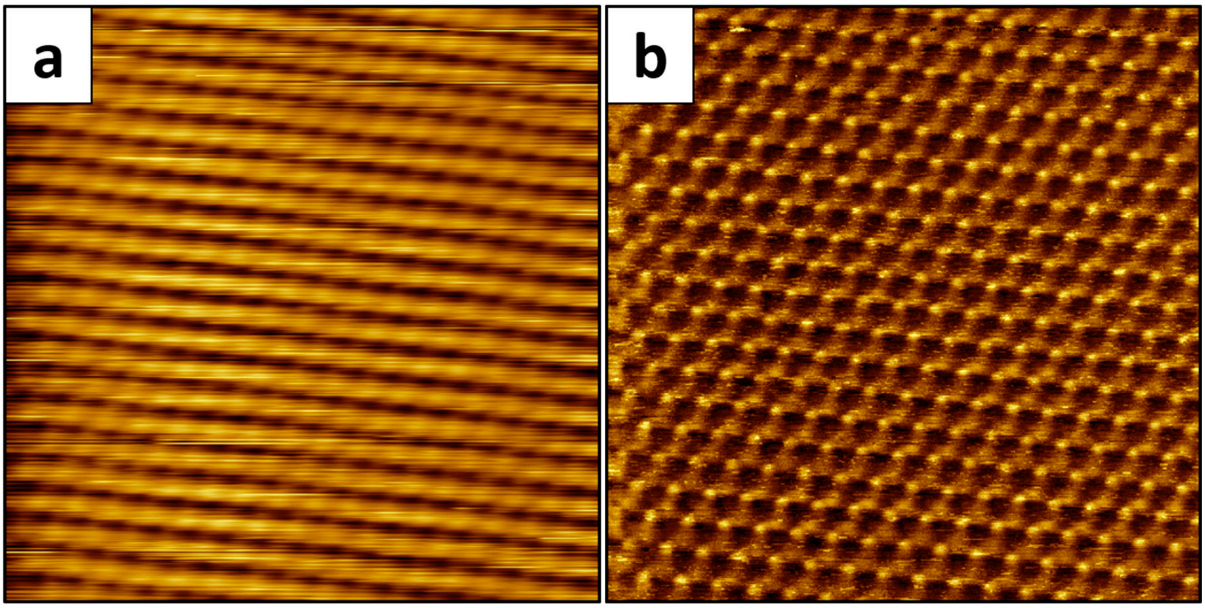

Figure S2. Atomic-resolution STM images (5 × 5 nm^2) obtained on a reconstruction-free island observed on HT-FeO/Ag(111) sample: Constant-current topography (**a**) and current map (**b**) (V = +0.1 V, I = 0.4 nA).

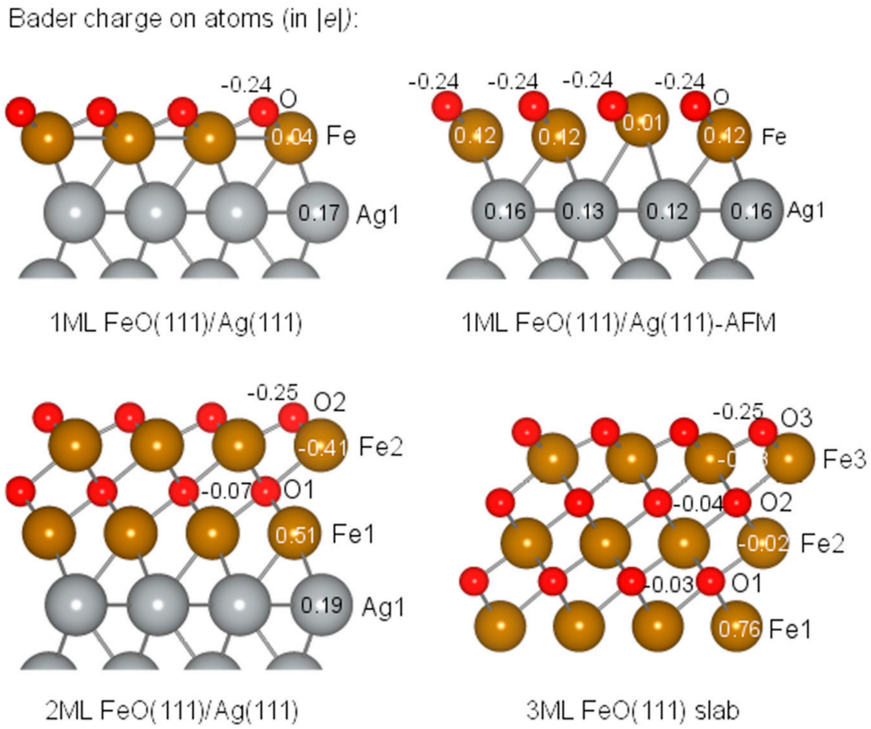

Figure S3. Graphical representation of the distribution of Bader charges on atoms calculated for different FeO systems (see SM4).

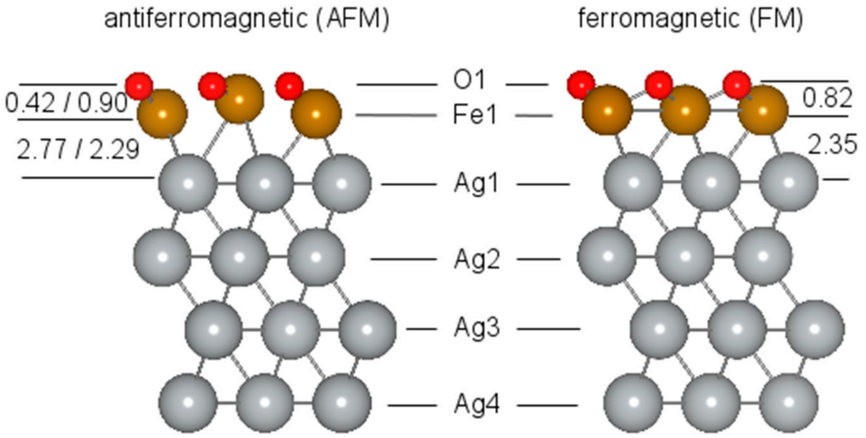

Figure S4. Differences in geometry of antiferromagnetic (AFM) and ferromagnetic (FM) superstructures of 1 ML FeO(111) on Ag(111). The AFM alignment of magnetic moments on Fe atoms makes the Fe layer distinctly rumpled, with Fe atoms of one direction of magnetization being by about 0.5 Å more distant from the Ag(111) surface than those with opposite magnetization. All distances are given in Å.
